# The COVID-19 pandemic and international federation of medical students’ association exchanges: thousands of students deprived of their clinical and research exchanges

**DOI:** 10.1080/10872981.2020.1783784

**Published:** 2020-06-19

**Authors:** Yassamine Bentata

**Affiliations:** a Department of Nephrology – Dialysis and Kidney Transplantation, Mohammed VI University Hospital, Oujda, Mohammed First University, Oujda, Morocco; b Laboratory of Epidemiology, Clinical Research and Public Health, Medical Faculty, Mohammed First University, Oujda, Morocco

**Keywords:** Covid-19 pandemic, medical students, clinical exchanges, research exchanges, deprivation

## Abstract

The repercussions of the COVID-19 pandemic for medical students are enormous and not limited to the interruption of courses in medical schools and/or hospital training rotations, the introduction of teaching exclusively online, postponement of examinations, and of the new academic year, but extend beyond that. The mobility of students within the framework of the International Federation of Medical Students’ Associations (IFMSA) is also strongly affected by the unexpected interruption of this program and the deprivation of thousands of students worldwide of this fine opportunity for training, exchange and sharing. The International Federation of Medical Students’ Associations (IFMSA) was founded in 1951 and currently maintains 135 National Member Organizations (NMOs) from 125 countries, representing a network of 1.3 million medical students around the globe. Moroccan students, members of IFMSA like students from other countries, are also concerned. Clinical and research exchanges, carried out under the auspices of the IFMSA, allow all the students to discover the health care and medical education systems of another country in a different sociocultural environment. To maintain student motivation and deal with the repercussions of cancelled exchanges, IFMSA could propose online training programs, adapted to student needs during this period of confinement and deconfinement from June to September 2020.

## Personal view

The repercussions of the COVID-19 pandemic for medical students are enormous and not limited to the interruption of courses in medical schools and/or hospital training rotations, the introduction of teaching exclusively online, postponement of examinations, and of the new academic year, but extend beyond that. The mobility of students within the framework of the International Federation of Medical Students’ Associations (IFMSA) is also strongly affected by the unexpected interruption of this program and the deprivation of thousands of students worldwide of this fine opportunity for training, exchange and sharing. IFMSA was founded in 1951 and currently maintains 135 National Member Organizations (NMOs) from 125 countries, representing a network of 1.3 million medical students around the globe (1). IFMSA is recognized as a non-governmental organization within the United Nations system and the World Health Organization and works in collaboration with world medical associations. It thus represents one of the world’s oldest and largest student-run organizations. Each year, more than 15,000 medical students embark on a journey to explore health care delivery and health systems in different cultural and social settings. An organization that annually, fulfills the dream of thousands of students, including those living in the most distant and most disadvantaged regions of the world. Students at all levels of medical studies are involved, from the first year to graduation with the degree in medicine. On average, these exchanges last four weeks and some may last eight weeks.

Very excited, motivated, and involved, students will leave their countries for several weeks, perhaps for the first time, heading to different destinations on five continents with the aim of learning, and discovering the functioning of other systems of health care and medical education. IFMSA-Morocco is the national member organization that has represented Morocco at IFMSA since 2012, year that the country officially joined this giant international student organization (2). However, it required two years of preparation and acquisition of the necessary documents, including the agreements and signatures of the deans of the faculties of medicine and the directors of university hospital centers of Morocco, before exchanges could really occur and membership take effect. The first exchanges from and to Morocco thus began in 2014/2015. It should be noted that the ‘academic year’ for the exchange program corresponds to the period between April of the year in progress and March of the following year. From the year 2014–2015 to the year −2019, 1,039 non-Moroccan students came to do 861 clinical exchanges and 178 research exchanges in Morocco. During this same period, 1,202 Moroccan students did 991 clinical and 211 research exchanges abroad. The Faculty of Medicine of Oujda, a city in the northeast of Morocco, received 106 foreign students of 25 different nationalities (60 in clinical and 46 in research exchanges) between the years 2015 and 2019, while 130 students left for 28 different destinations (98 for clinical and 32 for research exchanges).

The training programs through IFMSA provide an immense opportunity for Moroccan students to acquire new knowledge, develop new skills and consolidate acquired knowledge in two areas of medical studies, whether clinical or research. These exchanges are experiences that are not only very rich, rewarding, and interesting, but also marked by deep sharing and exchange between students of different nationalities and socio-cultural milieus. They also allow students to examine and participate in treatment of patients with diseases not usually encountered in their countries, such as malaria, brucellosis, typhoid, tuberculosis, leptospirosis, leishmaniasis, bilharziasis, hemorrhagic fevers, etc., diseases often studied in the lecture halls of the faculties or in medical textbooks, but rarely or never seen in hospital practice.

The students, in the framework of IFMSA exchanges, are received in hospital departments and/or affiliated research facilities, participate in different educational activities of the department, are integrated into the medical and paramedical teams, and accomplish predetermined objectives stipulated in their training handbooks, objectives that are appropriate to their level of study and type of exchange, clinical or research. Their educational progress is directly supervised by a senior educator of the hospital department or the director of a research facility. These exchanges also aim to develop more than the student’s medical and research abilities. Very important also for a young doctor, are the interpersonal, communication and management skills needed to communicate with all actors in the health system. Moreover, these exchanges provide an opportunity to strengthen language skills, and English is the universal language of communication between students of different nationalities. Students from Francophone countries may find difficulties in mastering English and language then becomes a real obstacle for some, limiting the choice of countries, and thus of exchange sites.

Beyond the educational and research goals, the students discover other cultural and social environments in a climate of respect, tolerance, sympathy and generosity. They are thus better prepared to approach and treat patients of foreign nationalities in their respective countries and develop ethical and cultural competencies accordingly. The activities of IFMSA are far from being limited to training programs, and extend to the organization of general assemblies, large international and regional events, medical prevention campaigns, and conferences.

The COVID-19 pandemic, triggered in December 2019, has affected more than seven million inhabitants in 218 countries, has immobilized air traffic in most of the affected countries, and has thus interrupted, across the world, the exchange program of students who should have started their assignments in April 2020. Thousands of students found themselves unable to take part in these exchanges, so desired and eagerly awaited. One part of these programs, notably those scheduled between August and March 2020, could resume if the land or air borders between countries were open and non-essential flights authorized. The exchanges that cannot be conducted this year will be postponed to next year, but this option deprives a great many students of the opportunity to participate.

To maintain student motivation and to make up for the exchanges cancelled because of the COVID-19 pandemic, IFMSA could propose special, adapted online training programs during the period of suspension, between June and September 2020, in the form of webinars, workshops, videos of medical simulation scenarios, and courses on diseases with active teaching methods and evaluation tools. Training on the acquisition of the fundamental bases of clinical research could also be organized in the same way as clinical training. It could also organize training sessions on student stress during this period of confinement and deconfinement. These types of training could be moderated by expert senior educators and co-moderated by IFMSA students. IFMSA could also organize, in collaboration with major accredited language centers and organizations, a rigorous, accelerated, certifying program to strengthen language skills, especially in English and Spanish, to benefit the students. IFMSA should play its supportive role for medical students during this period of the COVID-19 pandemic.

1. International Federation of Medical Students’ Associations. https://ifmsa.org/2020/05/26/climate-change-in-medical-curricula-a-need-for-current-and-future-physicians.
2. International Federation of Medical Students’ Associations- Morocco.

http://ifmsa-morocco.org

